# Mechanical Reinforcement in Nylon 6 Nanocomposite Fiber Incorporated with Dopamine Reduced Graphene Oxide

**DOI:** 10.3390/ma15155095

**Published:** 2022-07-22

**Authors:** Yonghuan Zhao, Yang Meng, Feichao Zhu, Juanjuan Su, Jian Han

**Affiliations:** 1 College of Materials and Textiles, Zhejiang Sci-Tech University, Hangzhou 310018, China; yhzhao33@163.com; 2 The Key Lab of Industrial Textile Material and Manufacturing Technology, Hangzhou 310018, China; m13357146889@163.com (Y.M.); zhufeichao@zstu.edu.cn (F.Z.)

**Keywords:** graphene oxide, polydopamine, PA6, composite fiber, reinforce, Young’s modulus

## Abstract

The emergence of graphene-based polymer composite fibers provides a new opportunity to study the high-performance and functional chemical fibers. In this work, we have developed an efficient and convenient method with polydopamine (PDA) to functionalize and reduce graphene oxide (GO) simultaneously, and the modified graphene nanosheets can obtain uniform dispersion and strong interfacial bonding in nylon 6 (PA6). Furthermore, the reinforced PA6 composite fibers were prepared through mixing PDA-rGO into the PA6 polymer matrix and then melt spinning. The functional modification was characterized by surface analysis and structural testing including SEM, TEM, FTIR, and Raman. When the addition amount of the modified GO was 0.15 wt%, the tensile strength and Young’s modulus of the composite fiber reached 310.4 MPa and 462.3 MPa, respectively. The results showed a meaningful reinforcement with an effect compared to the pure nylon 6 fiber. Moreover, the composite fiber also exhibited an improved crystallinity and thermal stability, as measured by DSC and TGA.

## 1. Introduction

It is an efficient and common route to prepare reinforced polymer nanocomposites by adding nanofillers. Extensive research work has reported that nanomaterials were used as reinforcing fillers such as Silica (SiO_2_), Montmorillonite (MMT), and Carbon Nanotubes (CNTs), which obtained ideal results in the application of polymer reinforcement [1,2,3]. Graphene and its derivatives, as one of the widely concerned carbon nanofillers, are two-dimensional materials composed of sp^2^-hybridized carbon atoms arranged in a honeycomb shape [4,5]. Its two-dimensional, sheet-like wrinkled structure provides an ultra-high specific surface area, and studies have confirmed it to be part of a promising new generation of nanofillers due to its outstanding mechanical properties and chemical stability [6]. Graphene can improve the mechanical, thermal, and electrical properties of polymers as a nanofiller, and it can also endow some polymer materials with flame retardant, anti-ultraviolet, antibacterial, and self-healing performances, among others [6,7]. Compared with traditional zero and one-dimensional fillers, graphene nanosheets exhibit significant advantages among the reinforcing fillers. It has been found that graphene and its derivatives can endow the polymer good reinforcing effect at a lower loading content [8]. This greatly expands the application prospects of graphene in the reinforcement of polymer composites.

Graphene nanosheets are prone to agglomeration in the polymer matrix because of the existence of van der Waals force [9,10]. Additionally, the poor interfacial characteristic between the graphene nanosheets and the polymer also leads to its restacking in the polymer matrix [11]. The main challenge is to achieve the effective dispersion and strong interface bonding of graphene nanosheet in polymer reinforced composites. In spite of that, nevertheless, the surface of graphene nanosheets is smooth without reactive functional groups. As one of the representative graphene derivatives, the surface of GO owns carboxyl, epoxy, and other active reaction functional groups onto the surface [4,6]. Surface modification of GO can effectively solve the dispersibility of graphene and its derivatives in polymers and the interfacial compatibility with polymers. Various surface modified ways of GO have been used in graphene/polymer composites through covalent bonds and non-covalent bonds [12,13,14].

In recent years, dopamine (DA) has received much attention because it can engage in self-polymerization to generate polydopamine (PDA) [15,16]. The strong interfacial adhesion property was demonstrated between the PDA layer and the matrix, including many substrates with low surface energy such as glass, ceramics, teflon, etc., which were attributed to the presence of reactive functional groups onto its surface [17,18]. The formed PDA surface possesses highly reactive functional groups, and it can be used to enhance the compatibility of nanofillers and polymers. Thus, DA has emerged as a functional modifier of nanofillers in the field of polymer reinforced nanocomposites [19,20,21,22]. Y.L. Lu et al. incorporated PDA-modified, multi-walled carbon nanotubes (MWCNTs) into natural rubber (NR) via latex compounding. The modified carbon nanotubes showed excellent dispersion in the matrix, increasing the tensile strength of the composite by 42% [20]. Y. Fang et al. prepared polyimide (PI) with polydopamine functionalization boron nitride nanosheets composite fiber by in situ polymerization, and the results indicated that the tensile strength and modulus of the composite fiber were a significant improvement with the addition of modified boron nitride nanosheets (0.5 wt% content) [21]. K.P. Chen et al. prepared TPU/PDA-GNP nanocomposite via in situ polymerization and discovered the strong interfacial interaction between PDA and TPU via covalent bonding, which led to a remarkable enhancement of the mechanical properties of the nanocomposite [22]. 

We attempted to modify the GO by dopamine self-polymerization on its surface, and GO can be reduced to PDA-rGO at the same time. Furthermore, the reinforced PA6 composite fibers were prepared by simple melt mixing and spinning. It is a simple, efficient, and low-cost method for the industrial production of polymer nanocomposite fibers. Herein, PA6 was selected as the polymer matrix of the fiber, while PA6 fiber was generally used for weaving various kinds of clothing materials and knitting, owing to excellent wear resistance and good resilience. As one of the widely used chemical fibers, it is also widely used in industrial textiles such as tire cords, fishing nets, and parachutes, which require high physical and mechanical properties [23,24]. Therefore, the PDA-rGO reinforced PA6 composite fiber provides a new path for its application in the field of high-performance industrial textile materials.

## 2. Experiment

### 2.1. Materials

PA6 slices (spinning grade, ρ = 1.13 g/cm^3^, MFR = 5.2 g/min at 250 °C) were supplied by Yiwu Huading Nylon Co., Ltd. (Jinhua, China); Graphite powder with particle size less than 20 μm was purchased from Doucheng chemical products trading Co., Ltd. (Tianjin, China); 98% Sulfuric acid (H_2_SO_4_), 37%Hydrochloric acid (HCl, L-Ascorbic acid (AR, 99%), Dopamine (DA), and Tris-HCl were provided from Aladdin Reagent Co., Ltd. (Shanghai, China); Sodium hydroxide (NaOH), 20% Hydrogen peroxide (H_2_O_2_), and 37% Hydrochloric acid (HCl) were purchased from Jiuding Reagent Co., Ltd. (Shanghai, China). 

### 2.2. Synthesis of Functionalized GO, rGO, and PDA-rGO 

**Figure 1:**
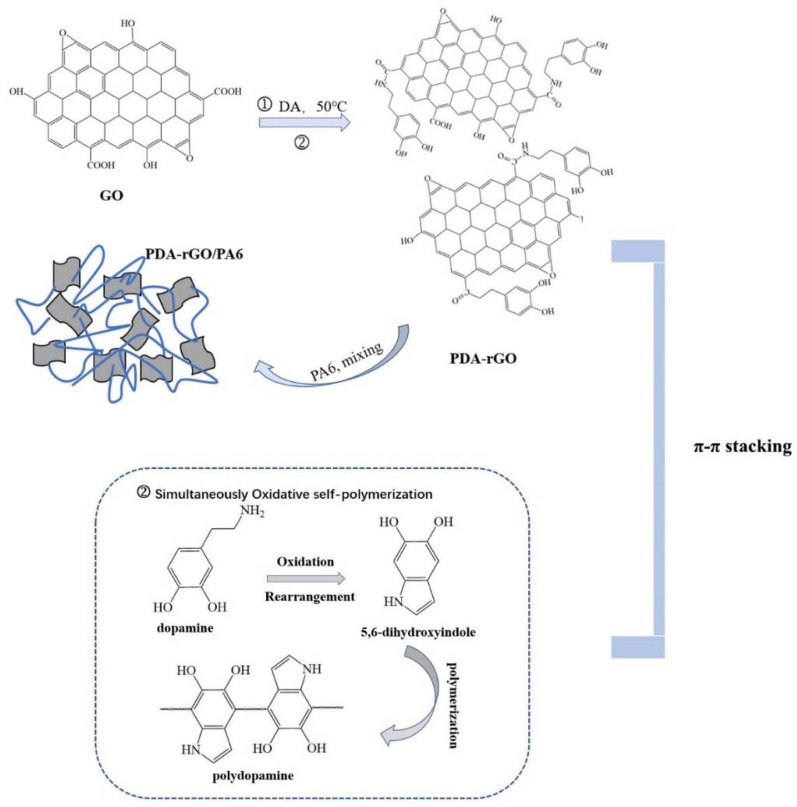
Schematic diagram of the synthesis process of PDA-rGO and PDA-rGO/PA6 composites.

Graphene oxide (GO) was prepared using a modified hummers method, as reported in our previous work [25]. The preparation of rGO (reduced Graphene Oxide) was followed by previous studies. The reduction reaction of GO was performed for 24 h at 80 °C, using L-ascorbic acid as the reducing agent. The process of synthesizing PDA-rGO (Polydopamine modified and reduced graphene oxide) is shown in Figure 1. First, 0.5 g GO was added to 500 mL deionized water and sonicated for half an hour to obtain a GO dispersion. Then, 0.64 g Tris-HCl was added to the GO dispersion and adjust the PH of solution to 8.5. Next, 20 mL (0.5 mg/mL) DA was added dropwise and reacted at 50 °C for 24 h. Finally, the resulting reactants were dialyzed and freeze-dried to obtain the powders of GO and PDA-rGO, respectively.

### 2.3. Preparation of PA6/-rGO and PA6/PDA-rGO Composite Fibers 

PA6 slices were dried in a vacuum oven at 100 °C for 24 h. The preparation process of the composite fibers was depicted in Figure1. PA6 composite fiber containing 0.05 wt%, 0.15 wt%, 0.3 wt% PDA-rGO was prepared using the twin screw extruder (MinLab) via melt blending method, as shown in Figure 1. The temperature of twin screw extrusion was 250 °C, and the speed was 50 rpm. The prepared primary fibers were then thermal drawn at 120 °C with a drawing ratio of 3.5. As a comparison sample, the composite fiber incorporated with 0.15 wt% unmodified rGO/PA6 was prepared under the same processing conditions.

### 2.4. Characterization 

The morphologies of GO, PDA-rGO, and PA6 composite fibers were observed using field emission scanning electron microscopy (SEM, vltra55). Transmission electron microscopy (TEM, JEM-2100) was used to record the microstructure of GO and PDA-rGO. In transmission mode, Fourier transform infrared spectroscopy (FTIR, Nicolet 5700) of GO, rGO, and PDA-rGO were tested with the scanning frequency 500~4000 cm^−1^. Raman spectroscopy (Raman, ThinVia) was used to evaluate structural changes before and after GO modification. The sedimentation experiments were provided a qualitative characterization of the dispersion properties. Next, 1 mg/mL nano-powder aqueous dispersion was prepared, and the settlement of the solution was observed after standing for 72 h. Thermal stability of GO, rGO, PDA-rGO, and the composite fibers were conducted on thermogravimetric analyzer (TGA, TG209F3). The heating rate of TGA test was 5 °C/min under nitrogen atmosphere. Mechanical properties of composite fibers were measured by a universal material stretching machine (6639, INSTRON), and the composite fibers (length, 25 mm) were measured at a tensile rate of 20 mm/min. Differential Scanning Calorimeter (DSC, Emerkin8000) was used to analyze the crystallization behavior of composite fibers. The heating and cooling rates were both 10 °C/min under nitrogen atmosphere.

## 3. Results and Discussion

### 3.1. The Morphology, Composition and Thermal Properties of PDA-rGO 

**Figure 2:**
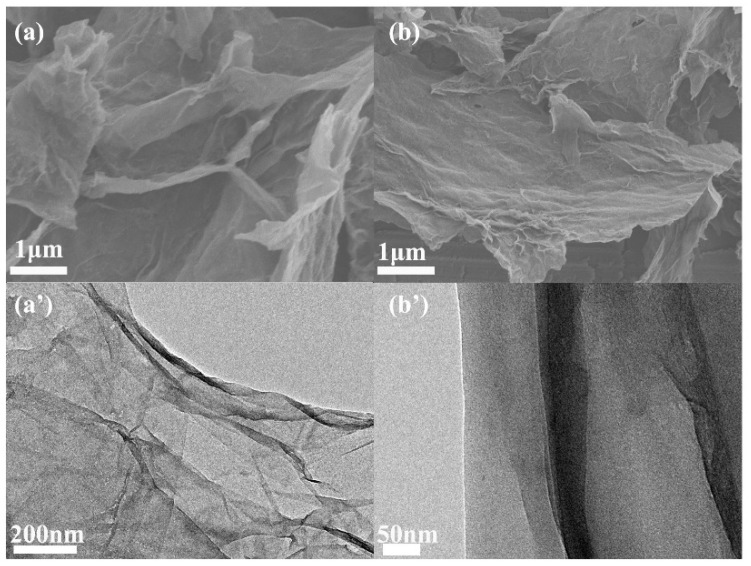
SEM images of GO (**a**) and PDA-rGO (**b**); TEM images GO (**a′**) and PDA-rGO (**b′**).

SEM and TEM images of the surface morphology and microstructure of GO and PDA-rGO are presented in Figure 2; GO has quite a multilayer sheet-like structure with a smooth surface and wrinkled edges, as shown in Figure 2a,a′. However, the surface of PDA-rGO is covered with a thin film after modification, as presented in Figure 2b,b′. This is the in situ polymerization of DA on the surface of GO under an alkaline environment, resulting in the formation of a uniformly wrapped PDA layer on the surface of GO [26]. It can also be seen that the PDA only wraps around the GO surface and does not change the multilayer sheet-like structure of GO.

**Figure 3:**
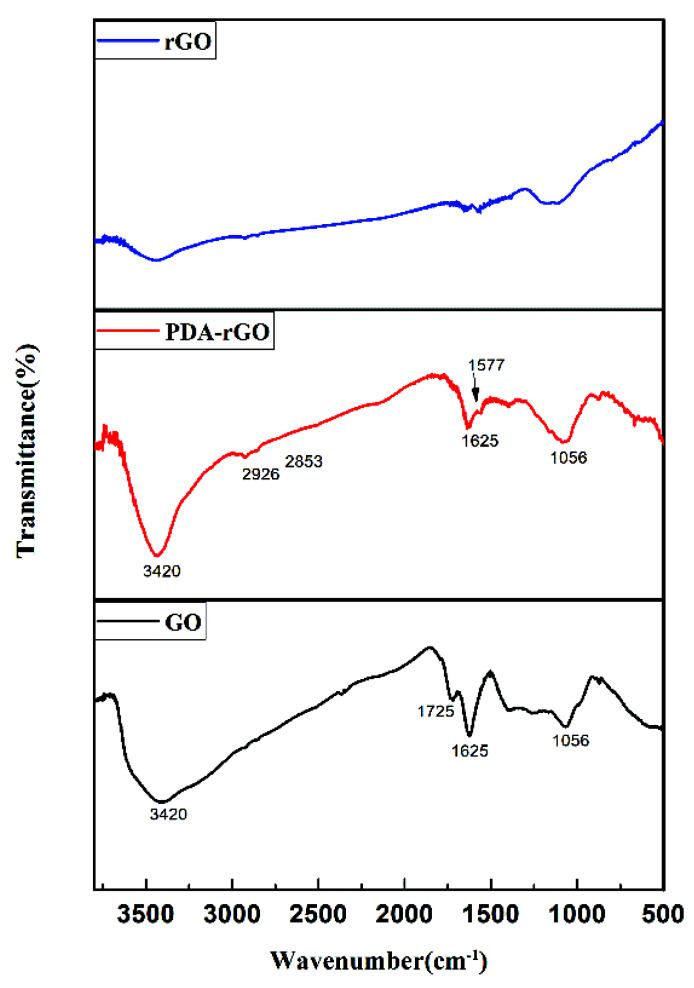
FTIR spectra of GO, rGO and PDA-rGO.

Figure 3 shows the FTIR spectra of GO, rGO, and PDA-rGO. The C=O stretching vibration peak at 1725 cm^−1^, the C-O stretching vibration peak of the hydroxyl group at 1056 cm^−1^, the C=C vibration peak of the aromatic ring at 1625 cm^−1^, and the stretching vibration peak of hydroxyl group O-H at 3420 cm^−1^ are the characteristic peaks of GO [19,25,27]. The C=O stretching vibration peaks of rGO and PDA-rGO at 1725 cm^−1^ decreased greatly, indicating that the carboxyl groups were mainly involved in the self-polymerization of dopamine onto the surface of GO. The newly generated peak at 1577 cm^−1^ is attributed to the N-H vibrational of PDA-rGO. The peaks of 2926 cm^−1^ and 2853 cm^−1^ can be ascribed to the symmetric stretching vibration and asymmetric stretching vibration of -CH_2_ in the molecular of PDA, respectively [22]. It can be confirmed that PDA was effectively coated onto the surface of GO through self-polymerization.

**Figure 4:**
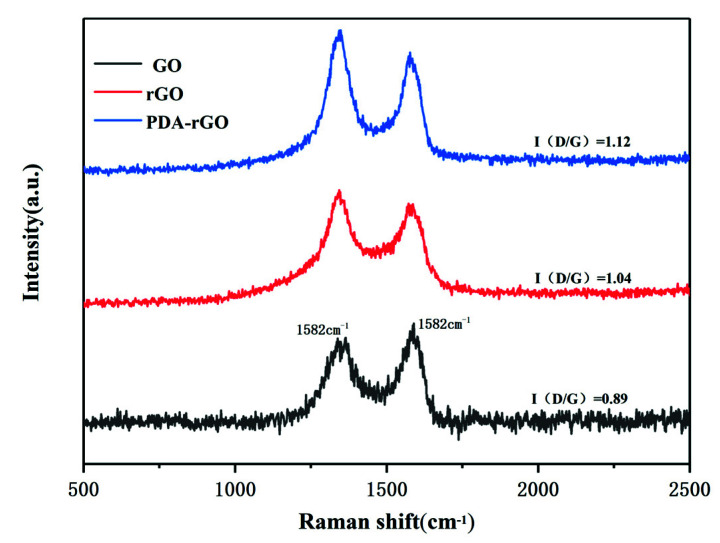
Raman spectra of GO, rGO and PDA−rGO.

The structures of GO, rGO, and PDA-rGO were further investigated using Raman spectra. As shown in Figure 4, all samples revealed two distinct absorption peaks around 1350 cm^−1^ and 1580 cm^−1^, namely the D band peak formed by the vibration of SP^3^ carbon atoms and the G band peak generated from the sp^2^ carbon atoms [27,28]. In most cases, the intensity ratio of the peaks (I_D_/I_G_, D band to G band) may be utilized to determine the degree of defects in the graphene structure [19,29]. The I_D_/I_G_ ratio of PDA-rGO was raised to 1.12, as compared to that of GO of 0.89. It demonstrated that PDA grafted on the surface of GO through the covalent bond, and thus its intensity ratio was higher than that of GO [27].

**Figure 5:**
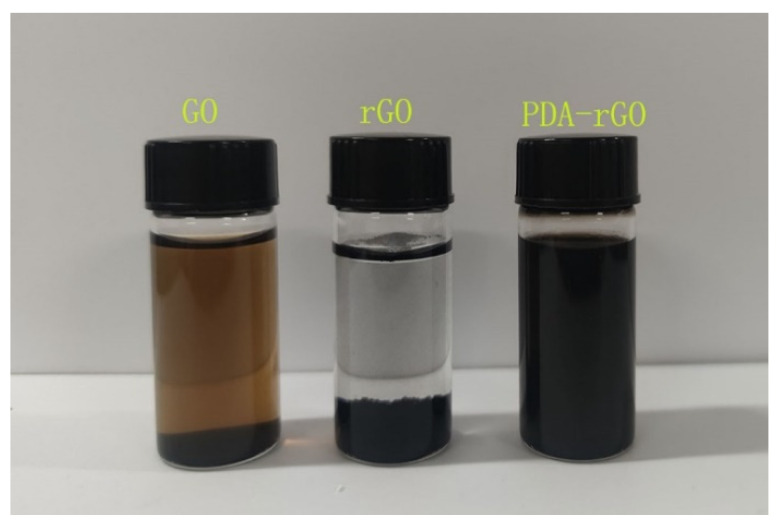
The optical photos of 1 mg/mL GO, rGO, and PDA-rGO solution after standing for 72 h.

The dispersion of carbon nanomaterials in aqueous solution can be investigated by sedimentation experiments. Figure 5 showed the optical photos of 1 mg/mL GO, rGO, and PDA-rGO after standing in aqueous solution for 72 h. GO exhibited good hydrophilicity due to the abundant oxygen-containing functional groups on the surface, therefore it can be uniformly dispersed in aqueous solution for a long time. The rGO produced a sedimentation phenomenon in the solution, while the PDA-rGO could still be stably dispersed in the aqueous solution because of the hydrophilic functional groups such as hydroxyl groups on its surface. In addition, the color of the aqueous solution in Figure 5 changed from brown to black, which was attributed to the removal of oxygen-containing functional groups and the recovery of aromatic ring π–π bonds [26]. It was further confirmed that graphene oxide was reduced by ascorbic acid and PDA.

**Figure 6:**
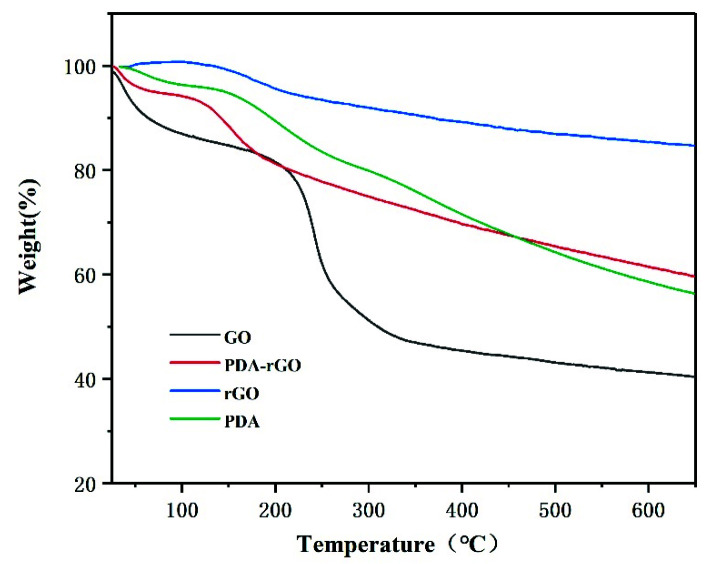
TGA curves of GO, rGO, PDA-rGO, and PDA.

Figure 6 presents the TGA plots of GO, rGO, PDA-rGO, and PDA. The weight loss of GO is substantially greater than that of rGO and PDA-rGO, below 120 °C, owing to the water and oxygen-containing groups on the surface of GO [30,31]. A major loss platform appears at around 200 °C~350 °C, and the weight loss rate of rGO and PDA-rGO is far less than that of GO. This result was attributed to the restore of the carbon skeleton structure of rGO and PDA-rGO resulting in the improved thermal stability [19]. The weight loss of GO, rGO, and PDA-rGO is 59.6%, 15.2%, and 40.2%, respectively, when the temperature reaches 600 °C. PDA exhibits better thermal stability compared to GO when the temperature is less than 400 degrees, while its maximum thermal decomposition temperature is 203.8 °C. After GO was modified by PDA through the covalent bond, the organic groups on the surface of PDA-rGO affected its thermal stability. In comparison with GO, the thermal stability of PDA-rGO was still effectively improved because the oxygen-containing functional groups on its surface are almost removed as a result of modification and reduction by PDA [22,26].

### 3.2. The Morphology of Composite Fibers

**Figure 7:**
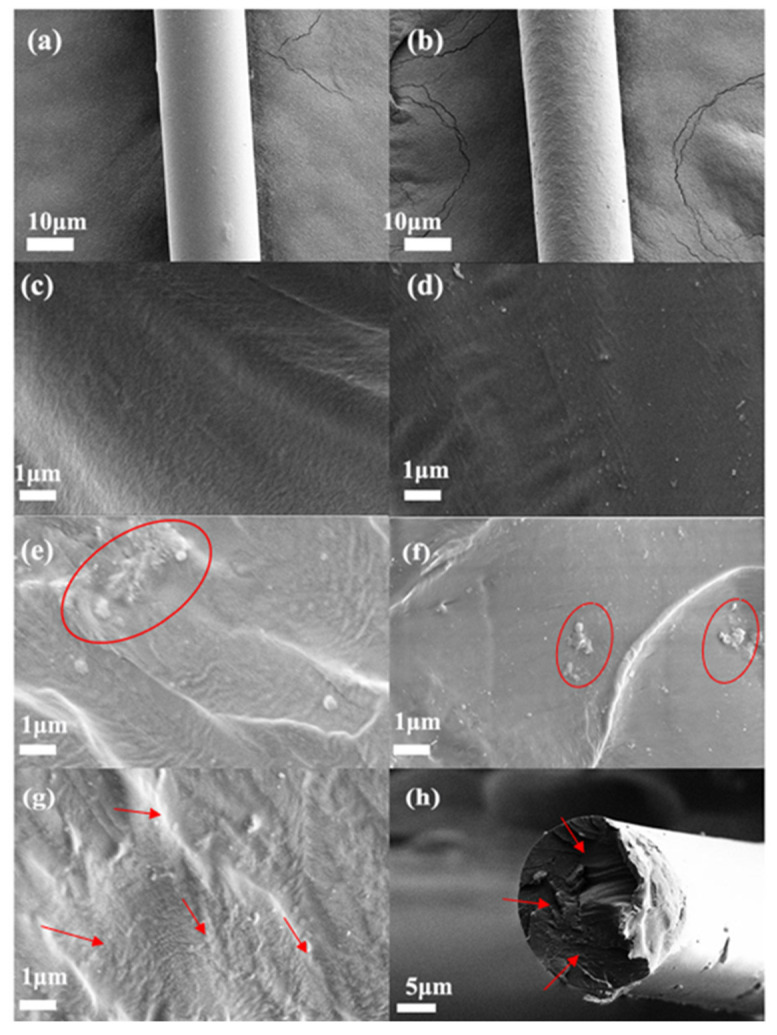
SEM images: the surface morphologies of PA6 fiber (**a**) and 0.15 wt% PDA-rGO/PA6 composite fiber (**b**); the cross-section morphologies of the fibers: PA6 (**c**), 0.05 wt% PDA-rGO/PA6 (**d**), 0.3 wt% PDA-rGO/PA6 (**e**), 0.15 wt%rGO/PA6 (**f**), and 0.15 wt% PDA-rGO/PA6 (**g**,**h**).

Figure 7 depicts the surface and cross-section morphology of the pure PA6 fiber and composite fibers. The surface of the pure PA6 fiber is relatively smooth and the fiber diameter is quite uniform, with an average value of 20 μm, as shown in Figure 7a. The surface roughness of the composite fiber increases with the addition of PDA-rGO, as shown in Figure 7b; however, the surface remains smooth and free of cracks and defects, demonstrating that the PDA-rGO/PA6 composite fiber can be spun and hot drawn continuously. The cross-sectional morphologies of the pure PA6 fiber, 0.15 wt% rGO/PA6 composite fibers, and various PDA-rGO/PA6 composite fibers are shown in Figure 7c–h. It can be seen from Figure 7f that the unmodified rGO clearly aggregates in the PA6 matrix because of the poor compatibility. When the PDA content reached 0.3 wt%, larger aggregates appeared in the composite fiber. On the other hand, 0.15 wt% PDA-rGO is nicely embedded and uniformly dispersed in the PA6 matrix, as shown in Figure 7g,h. Furthermore, neither holes or cracks are observed in the cross-section of the composite fiber, implying that the 0.15 wt% PDA-rGO and PA6 matrix has a robust interface interaction [32,33].

### 3.3. The Mechanical Properties of Composite Fibers

**Figure 8:**
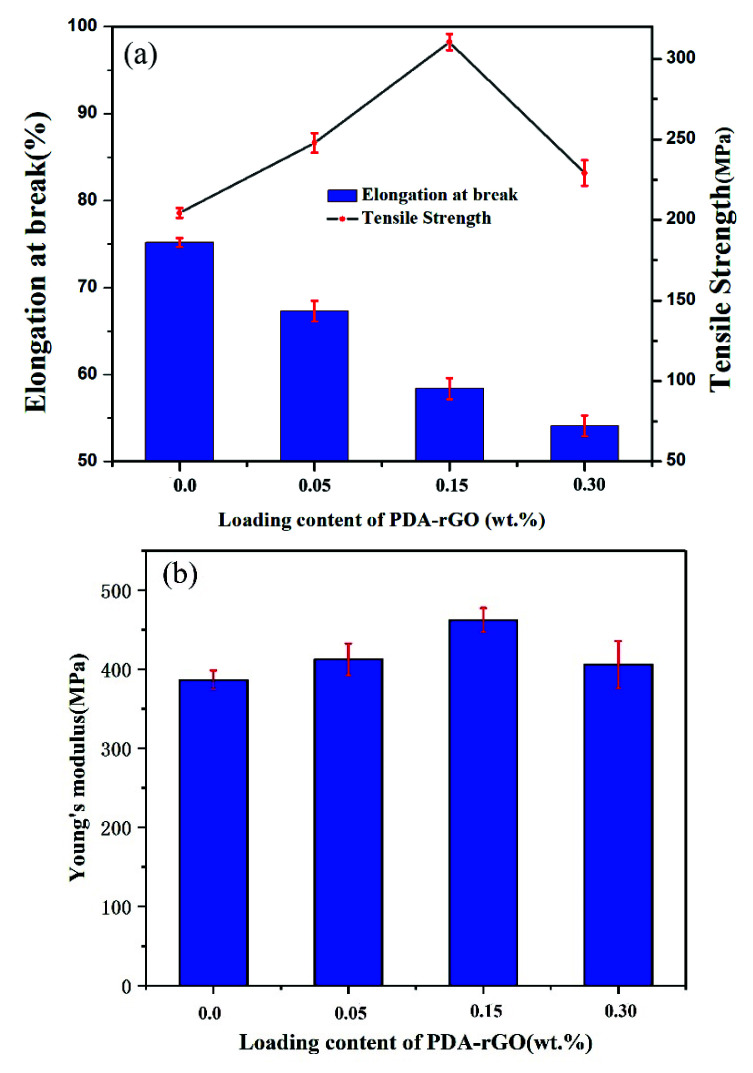
Strain–stress curves of PDA-rGO/PA6 composite fibers with various PDA-rGO content (**a**) and Young’s modulus (**b**).

The mechanical characteristics of the pure PA6 fiber, the PA6 composite fiber with 0.15% unmodified rGO, and the PDA-rGO/PA6 composite fibers with 0.05 wt%, 0.15 wt%, and 0.3 wt% PDA-rGO loading content are exhibited in Figure 8 and Table 1. The tensile strength and Young’s modulus (E) of 0.15 wt% rGO/PA6 composite fiber increases slightly compared with that of the pure PA6 fiber. The mechanical performance of the composite fiber is found to be significantly improved once PDA-rGO is added. The tensile strength and Young’s modulus of the PDA-rGO/PA6 composite fiber reaches a maximum value at 310.4 MPa and 462.3 MPa, respectively, when the PDA-rGO loading content is raised to 0.15 wt%. However, as the loading content further increases, the composite fiber’s tensile strength and Young’s modulus begin to decline owing to the excessive addition of PDA-rGO, which resulted in the production of flaws and weak joints on the composite fiber [32]. Comparing PDA-rGO/PA6 and rGO/PA6 composite fibers, it is found that the mechanical strength of the PDA-rGO/PA6 composite fiber increased by 45% over the rGO/PA6 composite fiber when the content is the same at 0.15 wt%.

**Table 1:** Tensile properties of PA6 fiber, 0.15 rGO/PA6 composite fiber and composite fibers containing different PDA-rGO content.

Loading Content (wt%)	Tensile Strength (MPa)	Elongation at Break (%)	Young’s Modulus (MPa)
0	204.3	75.2	386.4
0.05 PDA-rGO	247.8	67.3	412.3
0.15 PDA-rGO	310.4	58.4	462.3
0.3 PDA-rGO	229.1	54.1	405.8
0.15 rGO	213.5	46.5	394.6

The Halpin–Tsai model is one of the widely used polymer reinforcement mechanism models [19,34,35]. Herein, it is utilized to explore the reinforcement mechanism of PDA-rGO/PA6 composite fibers. In general, PDA-rGO may present ether an oriented or a random distribution state in the composite fibers. The theoretical Young’s modulus of the composite fibers is calculated by the following modified Halpin–Tsai equations:

(1) $$E_{r}\left(\mathrm{random}\right)=E_{N}\cdot \left[\frac{3}{8}\left(\frac{1+\eta_{L}\cdot \xi \cdot \nu_{G}}{1-\eta_{L}\cdot \upsilon_{G}}\right)+\frac{5}{8}\left(\frac{1+2\eta_{T}\cdot \upsilon_{G}}{1-\eta_{T}\cdot \nu_{G}}\right)\right]$$

(2) $$E_{P}\left(\mathrm{parallel}\right)=E_{N}\cdot \left(\frac{1+\eta_{L}\cdot \xi \cdot \upsilon_{G}}{1-\eta_{L}\cdot \upsilon_{G}}\right)$$

(3) $$\eta_{L}=\frac{E_{G}/E_{N}-1}{E_{G}/E_{N}+\xi }$$

(4) $$\eta_{G}=\frac{E_{G}/E_{N}-1}{E_{G}/E_{N}+2}$$

(5) $$\xi =\frac{l+\omega }{d}$$

where E_r_ and E_p_ denote Young’s modulus of PDA-rGO/PA6 composite fibers with random and parallel oriented PDA-rGO nanosheets. E_N_ represents the Young’s modulus of pure PA6 fiber (386.4 MPa), while E_G_ refers to the modulus of PDA-rGO (250 GPa) [19,34]. In this model, the modified graphene nanosheets are regarded as strip solid fibers, with l, ω, and d representing the length, width, and thickness of PDA-rGO (l = 3 μm, ω = d 2 μm, d = 2.4 nm), respectively [35]. The mass fraction of PDA-rGO used in the experimental section is converted into volume fraction V_G_ (%) from the following equations:

(6) $$V_{G}\left(\%\right)=\frac{W_{G}/\rho_{G}}{W_{G}/\rho_{G}+\left(1-W_{G}\right)/\rho_{N}}$$

where W_G_ is the mass fraction of PDA-rGO in the PA6 matrix, ρ_G_ (1.8 g/cm^3^) and ρ_N_ (1.13 g/cm^3^) denote the density of PDA-rGO and pure PA6 fiber, respectively [19,35].

**Figure 9:**
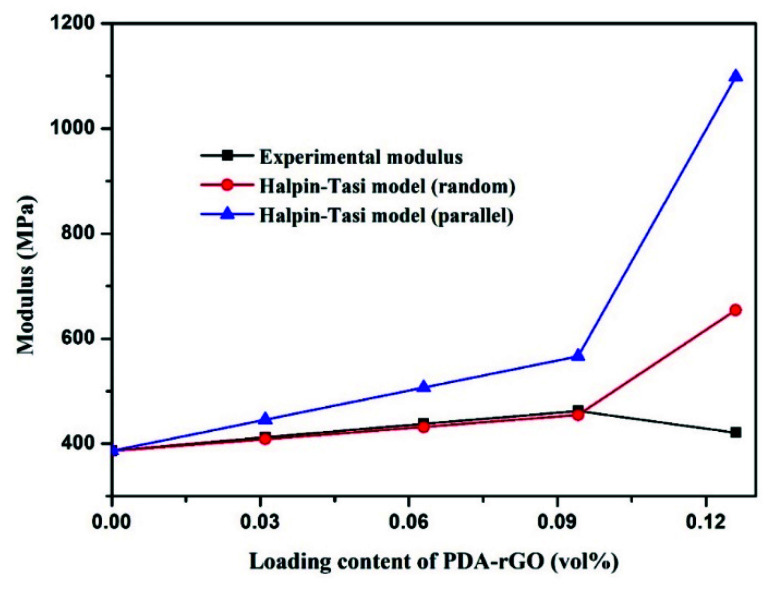
Experimental Young’s modulus data and theoretical Young’s modulus obtained by the Halpin–Tsai model for PDA-rGO/PA6 composite fibers.

Figure 9 shows the experimental modulus and the Halpin–Tsai theoretical modulus of the PDA-rGO/PA6 composite fiber. When the volume fraction of PDA-rGO is less than 0.1%, the experimentally measured Young’s modulus data are pretty much close to theoretical calculated value by the random distribution model of PDA-rGO in PA6 matrix, and they both show an upward trend with an increase in filler content. This indicates, on the one hand, that PDA-rGO is randomly distributed in the matrix, and, on the other hand, that at, low contents, the experimental and theoretical values basically coincide because PDA-rGO can be uniformly dispersed in the matrix without causing stress concentration due to agglomeration in the matrix. Similar findings were reported by Liu Haihui, Jia Hongbing, etc., [19,35,36]. When the volume fraction of PDA-rGO exceeds 0.09%, however, the experimental modulus deviates from the value of theoretical model and shows a downward trend. This is primarily due to the agglomerates of PDA-rGO in the matrix, which may introduce defects and thus become a mechanical weak point.

At this point, we can rule out the contribution of PDA-rGO orientation to the mechanical properties, and the reason directed at the non-orientation is probably related to the fact that the tensile stress field applied by our processing equipment is not that strong. The following aspects are accountable for the substantial improvement in the mechanical properties: (i) the PDA-modified graphene nanosheets can be uniformly dispersed in the PA6 matrix, which not only greatly avoids the stress concentration caused by agglomeration, but also brings out the laminar fold structure of rGO as much as possible, playing a much better role in stress dispersion and transfer [37]; (ii) there is a good interfacial compatibility and strong interfacial bonding between the PDA-rGO and PA6 matrix, which, on the one hand, can prevent the slip of the matrix molecular chain on the PDA-rGO surface, and can also effectively transfer the stress to the PDA-rGO; (iii) the molecules on the surface of PDA-rGO and the PA6 macromolecular chain may form a certain degree of three-dimensional network structures through hydrogen bond interaction and entanglement during the melt mixing and spinning process. It restricts the movement of macromolecular chains, which also explains the gradual decrease in the elongation at break of the composite fibers [11,32].

### 3.4. The Thermal Properties of Composite Fibers

The crystallization behavior of PA6 fiber and composite fibers can be measured by DSC. Here, T_m_ (the melting temperature) and T_c_ (the crystallization temperature) were tested as the peak points. $X_{c}\;$(crystallinity) was measured by the following formula:

(7) $$X_{c}=\frac{{\Delta H}_{f}}{\left(1-\alpha \right){\Delta H}_{f^{*}}}\times 100\%$$

where *α* represents the proportion of PDA-rGO filler in the PA6 matrix, ΔH*_f_* refers to the melting enthalpy of the prepared composite fibers, and ΔH_f*_ is the melting enthalpy of 100% crystallization of PA6 corresponding to190 J/g.

**Figure 10:**
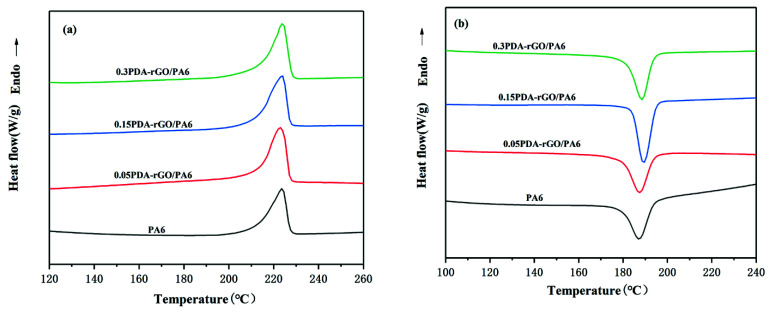
DSC plots of PDA-rGO/PA6 composite fibers with various PDA-rGO content: (**a**) melting curves; (**b**) crystallization curves.

T_m_ of the PDA-rRG/PA6 composite fibers does not change much with the incorporation of PDA-rGO, as shown in Figure 10 and Table 2, while T_c_ moves toward higher temperature. In addition, the crystallinity increases slightly up to 33.3% when the PDA-rGO content is 0.15%. Because of its homogeneous dispersion in the PA6 matrix, PDA-rGO may act as a heterogeneous nucleating agent, promoting crystallization of PA6 [38]. The crystallinity of the composite fibers starts to decline inversely after the addition of 0.3% PDA-rGO. This is due to the reduced nucleation efficiency, since the excess graphene nanosheets may agglomerate and accumulate in PA6. As can be observed, the effect of PDA-rGO on the crystallization behavior of the composite fibers is not significant. This may be due to the thermal stretching field experienced in the fiber preparation, where the stresses ordered the PA6 molecular chains and promoted the crystallization, thus the effect of PDA-rGO on the crystallization of the matrix is weakened and not to be manifested [37]. It also suggests that crystallinity should have little to do with the above mechanical properties.

**Figure 11:**
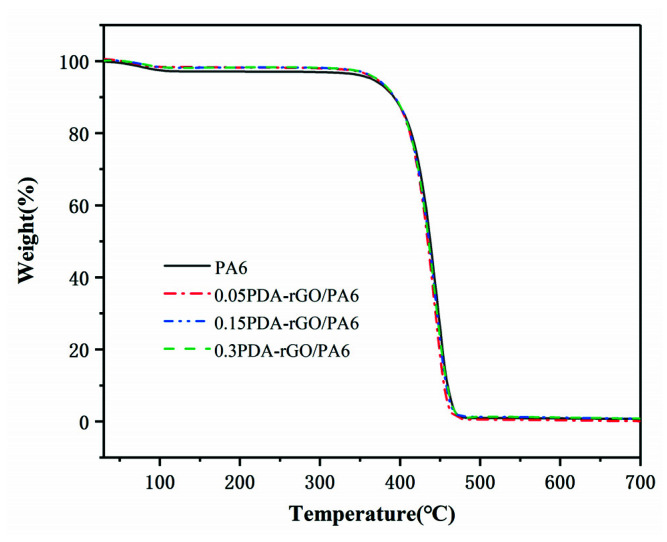
TGA curves of PDA-rGO/PA6 composite fibers with different PDA-rGO content.

The TGA results of pure PA6 and composite fibers are shown in Figure 11, meanwhile, the specific data of T_5_ (the decomposition temperature at 5% weight loss) and T_max_ (the decomposition temperature at maximum weight loss) are listed in Table 3. Compared with pure PA6 fiber, T_5_ of the composite fiber increases by 13 °C to 364.3 °C when the PDA-rGO loading content is 0.15 wt%, and the T_max_ decomposition occurs at 445.7 °C. The addition of PDA-rGO significantly improves the thermal stability of composite fibers. The improved thermal stability of the composite fiber is assisted by the uniform dispersion of modified graphite nanosheets in the matrix, which can form a three-dimensional network structure that will restrict the molecular movement of the PA6 matrix as well as a certain physical barrier effect that suppresses the thermal decomposition of the composite fibers [32,39].

## 4. Conclusions

In summary, the reinforced PDA-rGO/PA6 composite fibers with 3D network structure were prepared by simple melt mixing. PDA was successfully grafted on the surface of GO through covalent bond, and the modification and reduction of GO were completed simultaneously. At a loading content of 0.15 wt% PDA-rGO, the PDA-rGO/PA6 composite fiber displayed significantly improved tensile strength (310.4 MPa) and Young’s modulus (462.3 MPa). The DSC results revealed that the crystallinity increased from 30% to 33% due to the heterogeneous nucleation of PDA-rGO, and the thermal stability was also improved. The good dispersibility and enhanced interfacial interaction endowed the composite fibers with remarkable mechanical properties. Therefore, modified graphene composite fibers provide a new and improved way to prepare reinforcing fibers.

## Figures and Tables

**Table 2:** DSC data of PA6 fiber and composite fibers containing different PDA-rGO content.

Loading Content (wt%)	T_m_ (°C)	ΔH*_f_* (J/g)	T_c_ (°C)	*X_c_* (%)
0	223.6	54.3	187.4	28.6
0.05 PDA-rGO	223.0	58.6	187.5	31
0.15 PDA-rGO	223.8	63.2	189.2	33.3
0.3 PDA-rGO	223.4	59.8	188.6	31.6

**Table 3:** TGA data of PA6 fiber and composite fibers containing different PDA-rGO content.

Loading Content (wt%)	T_5_ (°C)	T_max_ (°C)
0	351.3	440.5
0.05 PDA-rGO	355.8	443.8
0.15 PDA-rGO	364.3	445.7
0.3 PDA-rGO	363.9	442.1
